# Ethical Issues of Digital Twins for Personalized Health Care Service: Preliminary Mapping Study

**DOI:** 10.2196/33081

**Published:** 2022-01-31

**Authors:** Pei-hua Huang, Ki-hun Kim, Maartje Schermer

**Affiliations:** 1 Department of Medical Ethics, Philosophy and History of Medicine Erasmus MC University Medical Center Rotterdam Netherlands; 2 Department of Industrial Engineering Pusan National University Busan Republic of Korea

**Keywords:** digital twins, digital health, personalized health care service, data-driven health care, value-sensitive design, ethics of health care technology

## Abstract

**Background:**

The concept of digital twins has great potential for transforming the existing health care system by making it more personalized. As a convergence of health care, artificial intelligence, and information and communication technologies, personalized health care services that are developed under the concept of digital twins raise a myriad of ethical issues. Although some of the ethical issues are known to researchers working on digital health and personalized medicine, currently, there is no comprehensive review that maps the major ethical risks of digital twins for personalized health care services.

**Objective:**

This study aims to fill the research gap by identifying the major ethical risks of digital twins for personalized health care services. We first propose a working definition for digital twins for personalized health care services to facilitate future discussions on the ethical issues related to these emerging digital health services. We then develop a process-oriented ethical map to identify the major ethical risks in each of the different data processing phases.

**Methods:**

We resorted to the literature on eHealth, personalized medicine, precision medicine, and information engineering to identify potential issues and developed a *process-oriented ethical map* to structure the inquiry in a more systematic way. The *ethical map* allows us to see how each of the major ethical concerns emerges during the process of transforming raw data into valuable information. Developers of a digital twin for personalized health care service may use this map to identify ethical risks during the development stage in a more systematic way and can proactively address them.

**Results:**

This paper provides a working definition of digital twins for personalized health care services by identifying 3 features that distinguish the new application from other eHealth services. On the basis of the working definition, this paper further layouts 10 major operational problems and the corresponding ethical risks.

**Conclusions:**

It is challenging to address all the major ethical risks that a digital twin for a personalized health care service might encounter proactively without a conceptual map at hand. The process-oriented ethical map we propose here can assist the developers of digital twins for personalized health care services in analyzing ethical risks in a more systematic manner.

## Introduction

### Background

The concept of digital twins is expected to transform the landscape of existing health care systems [1,2]. Originating from industrial design, the concept of digital twins capitalizes on the data of specific objects to simulate replicas in the virtual world for predictive analysis of safety risks and testing of different optimization solutions. Applying to the health care sector, a digital twin can be a virtual replica of a particular patient that reflects the unique genetic makeup of the patient [3] or a simulated 3D model that exhibits the characteristics of a patient’s heart [4,5]. Ideally, these digital twins will allow clinicians to offer personalized health care to individual patients.

Digital twins may make health care services more proactive and personalized. With predictive algorithms and real-time data, digital twins have the potential to detect anomalies and assess health risks before a disease develops or becomes symptomatic. Information provided by digital twins can then help clinicians determine whether early intervention is necessary [6,7]. Digital twins equipped with data such as patients’ genetic information and disease history may also facilitate clinicians to *personalize* the treatment [8,9]. Each individual has a unique genetic makeup, experiences different diseases, and lives in a different environment. These differences also make individual patients respond to different treatments in different ways. Under certain circumstances, the response could be so adverse that the treatment meant to heal the patient causes hospitalization or even death. It is envisioned that in the future, clinicians may simulate the effects of all possible treatments on patients’ digital twins first and determine which option is likely to be the most effective for the patients.

The idea of digital twins for personalized health care is gaining traction from both the public and private sectors. Several research consortia have been established to explore the potential of digital twins for personalized health care services. In the Netherlands, the Erasmus MC University Medical Center, Delft University of Technology, and Erasmus University Rotterdam have jointly initiated research on digital twins and cardiovascular disease prevention [10]. Sweden’s Linköping University also launched a pioneering research project, MeDigiT [11], to explore the potential of digital twins for medical education, heart disease diagnosis, medical implant planning, and so forth. Another 2 major research groups in Europe, the Swedish Digital Twin Consortium [12] and the DigiTwins [13], focus on translating the concept of digital twins into fields such as molecular medicine and genomic research. In the private sector, Philips has rolled out a clinical application called HeartModel that allows cardiologists to plan their upcoming surgeries with high-resolution interactive 3D models that reflect the distinctive and unique features of their patients’ hearts [4]. Siemens Healthineers and GE Healthcare also have similar products under development [5].

However, the growing interest in applying the concept of digital twins to personalized health care has raised new questions. Indeed, the concept of a digital twin is well-established and widely applied in industrial design and engineering. There is consensus about what sort of data and infrastructure are required to develop a digital twin for purposes such as predictive maintenance and optimization planning [7]. Nonetheless, the *objects* involved in the health care sector are very different from those in the realm of engineering. Even with the completion of the Human Genome Project, our understanding of genes and diseases is still very limited. Developing a digital twin that can adequately simulate or predict a person’s health condition is much more challenging than building a digital replica of a nonliving object. In addition, humans are living beings with personal commitments and moral worldviews. Applying the engineering concept to health care without taking these differences into account will be deeply problematic.

To our knowledge, there is currently no comprehensive review on the ethical risks of developing personalized health care services based on the concept of digital twins. Indeed, some have already noted that the data-driven nature of digital twins requires developers to pay special care to privacy protection [14]. However, privacy is only one of the ethical risks developers of digital twins need to carefully address during development. The involvement of predictive algorithms, for instance, could expose users to algorithmic biases [15]. Bruynseels et al [16] also argue that digital twins might worsen existing inequalities. Those who are less well-off might not have the means to take advantage of the service because of a lack of access to devices that can precollect the data required for using the service. Given the ever-increasing interest in transforming the health care sector with the concept of digital twins, it is urgent to identify the major ethical risks of digital twins for personalized health care services.

### Objectives

This research aims to address this research gap by providing a comprehensive analysis of the major ethical risks of digital twins for personalized health care services. Owing to the scarcity of literature on digital twins for personalized health care services, we are unable to perform a systematic review of ethical concerns over digital twins for personalized health care services. As a convergence of health care, artificial intelligence, information and communication technologies (ICTs), and personalized health care services, it is also difficult to apply the existing bioethical framework to capture the distinctive features and corresponding ethical risks of digital twins for personalized health care services. In addition, as influential bioethical frameworks such as the Beauchamp and Childress [17] 4 principles framework focus on high-level abstract ethical principles, it is challenging to translate the principles into specific and concrete normative guidance for first-line developers.

## Methods

Owing to the challenges stated in the *Introduction* section, we resorted to the literature on eHealth, personalized medicine, precision medicine, and information engineering to identify potential issues. We developed a *process-oriented ethical map* to structure the inquiry in a more systematic way. The *ethical map* allows us to see how each of the major ethical concerns emerges during the process of transforming raw data into valuable information. Developers of a digital twin for personalized health care service may use this map to identify ethical risks during the development stage in a more systematic way and proactively address them.

We first provided a working definition of digital twins for personalized health care services to clarify the extent to which our research was applicable. We then consulted the literature on ICTs to develop a process-oriented ethical map to structure the identified ethical risks. We have discussed the limitations of our research and provided recommendations at the end of this paper.

## Results

### Digital Twins for Personalized Health Care Service: Working Definition

#### Overview

Despite all the interest in translating the concept of digital twins to personalized health care services, there is *no* consensus on the definition of digital twins for personalized health care services. Some services focus on visualization, whereas others aim to offer predictive analysis. Some capitalize on existing data, whereas others require continuous input. Differences such as these create challenges in providing a systematic analysis of potential ethical pitfalls of digital twins for personalized health care service.

The lack of consensus is attributable to 2 reasons. The first reason is the ambiguity of *personalization* [15]. Each patient is different in various aspects. Every individual has different molecular and genomic features, and no patient has the same socioeconomic background, preferences, needs, and conception of the good. Therefore, personalized health care could be understood in at least two ways. When focusing on the biological aspect, the meaning of personalization is akin to precision. Following this interpretation of personalization, the general goal of personalized health care is to fine-tune health care with health-related data and administer treatments that are likely to be most effective and cause the fewest adverse side effects to a patient. Achieving personalization in this sense does not require active participation from the patient. In contrast, when focusing on the nonbiological aspect, to realize personalization is to respect individual patients’ personal commitments and values. Personalized health care, in this sense, is a health care ideal that aims to give back agency to the patient, facilitating the patient to autonomously choose the treatment course that can best reflect their values or cater to their particular needs. Thus, the improvement of the patient’s physical health would not be the primary consideration here.

The second reason is the difference in goals. Each personalized health care service aims to address different health issues. The solutions adopted by developers might also vary. Phillip’s HeartModel provides a personalized health care service in the sense that the digital twin reflects the unique anatomical structure of a particular patient. Their goal was to improve the quality of the surgery. As for My Digital Twin, developed by the Dutch research team, the goal was to use a digital twin to crunch health-related data to predict whether a person was on the trajectory of developing cardiovascular diseases. Although the 2 cases shared a general goal, that is, improving health care quality, the health issues they aimed to address and the services they aimed to provide were very different.

In this study, we do not aim to provide a definitive account of what a digital twin for personalized health care services ought to be. However, to begin a critical analysis of potential ethical pitfalls, we propose a working definition to clarify the extent to which our ethical framework is applicable.

#### Definition of a Digital Twin for Personalized Health Care Service

A digital twin for personalized health care service is a *data-driven, interactive computerized model* that aims to offer *health-related information* that properly simulates or predicts the health conditions of a *particular person*.

#### Data-Driven, Interactive Computerized Model

Any attempt to incorporate the concept of digital twins in a health care system requires an input of data. The general idea behind the concept of digital twins is to capitalize on precollected or real-time data to build up interactive models that allow users to conduct various simulations (eg, descriptive modeling, predictive analysis of risk levels, or prescriptive recommendation). In the health care context, the general goal of a digital twin for personalized health care service is to capitalize on data that are directly or indirectly related to an individual patient’s health conditions to build up computerized models that allow users (the patient or relevant clinicians) to gain an opportunity to devise and test different virtual trials (eg, lifestyles, pharmaceutical interventions, and surgical approaches) on the patient’s digital twin. The data used by a digital twin for personalized health care service can be identifiable data or nonidentifiable data. For instance, a digital twin’s predictive algorithms can be trained with multiple deidentified data sets and can make predictions on a person’s health trajectory based on certain identifiable data provided by the person.

#### Health-Related Information

Data treated properly can yield 3 types of health-related information [18]. *Descriptive information* indicates what has happened or is happening to a person’s health. *Predictive information* offers insights regarding what is likely to happen to a person’s health. *Prescriptive information* provides suggestions regarding which action or intervention should be adopted for the sake of improving or restoring a person’s health. These 3 information types are essential for personalized health monitoring, diagnosis, prognosis, prevention, and treatment.

Depending on the goals, the information provided by a digital twin for personalized health care service may involve only 1 type of information or multiple types of information. A simple 3D model of a patient’s heart, for instance, might only deliver descriptive information. In contrast, a model built from a patient’s genomic data might offer more than a mere description of the patient’s health. For instance, a digital twin built from a person’s genomic data has the potential to predict the effectiveness of a particular treatment course for a specific patient and prescribe treatment recommendations for a specific patient.

#### Particular Person

The computerized model or prediction generated by a digital twin must properly simulate the unique characteristics of a person. In this sense, incorporating the concept of a digital twin into the health care sector may yield a new form of personalized health care service.

However, the focus on simulating the health conditions of a particular person does not mean that a digital twin for personalized health care services can only use data from the person. Calibration of the algorithms used for simulation or prediction may require a large amount of health-related data collected from the general public. For instance, providing more personalized advice regarding blood pressure and hypertension management will require the developers to first work with relevant data to refine the baseline blood pressure.

This working definition helps us differentiate a digital twin for personalized health care service from general digital health care (or eHealth). For instance, although telehealth also capitalizes on ICTs and arguably requires data input from the patient (eg, via teleconsultation), telehealth services do not depend on computerized modeling. Instead, the value of telehealth results mainly from offering patients the opportunity to consult their clinicians remotely. The requirement of interactability also helps us distance a digital twin for personalized health care service from medical technologies that have long been adopted to create digital images of particular persons. For instance, although magnetic resonance imaging (MRI) also relies heavily on ICTs to transform the collected data into imagery information of specific persons, these computerized images do not offer clinicians the opportunities to conduct virtual trials on them. As a result, MRI does not qualify as a digital twin for personalized health care service but is a medical device that may be incorporated into a digital twin for personalized health care service. One of Linköping University’s MeDigiT projects, for instance, uses MRI and computed tomography data to simulate a heart digital twin to better personalize the artificial heart implant (Table 1).

**Table 1:** Examples of different types of digital twins for personalized health care service.

Type of digital twin	Type of primary data used by the digital twin	Forms of digital twin	Types of information delivered
HeartModel [4]	Imagery data (ultrasound)	Interactive visual presentation of the anatomical and physiological features of the heart	Descriptive
MeDigiT [11]	Imagery data (MRI^a^ and CT^b^)	Interactive visual presentation of the anatomical and physiological features of a specific part of the body	Descriptive
My Digital Twin [10]	Lifestyle data (dietary, smoking, use of alcohol, and medication), environmental data (living and working situations), and electronic health records (visits to health care services, medication, biotest results, MRI scans, and CT scans)	An aggregated model that offers information about a person’s current health conditions, a prediction of relevant health risks, and health advice on improving the health condition	Descriptive, predictive, and prescriptive
Personalized diagnosis and therapy via genomic medicine [3]	Genomic data	A genomic model allows users to identify treatments that are likely to be most effective for a particular patient	Predictive and prescriptive

^a^MRI: magnetic resonance imaging.

^b^CT: computed tomography.

### A Process-Oriented Ethical Map

#### Overview

Digital twins for personalized health care services aim to synthesize valuable information from health-related data for timely diagnosis, prognosis, preventive intervention, or treatment optimization. Achieving these goals requires a sophisticated orchestration of multiple ICTs and the involvement of various stakeholders in tasks such as data collection, data analysis, and information presentation. Each of the data processing phases faces different ethical risks. It is challenging to address all the major ethical risks that a digital twin for personalized health care service might encounter proactively without a conceptual map at hand. The process-oriented ethical map we have proposed below would assist developers of digital twins for personalized health care services in analyzing ethical risks in a more systematic manner (Table 2).

Briefly, despite the complex infrastructure, the process of creating valuable information can be divided into four major phases: data collection, data management, data analysis, and information use. Each of the 4 major phases requires different ICTs and information systems to realize its desired goal. It is not surprising that each of the 4 phases touches on various ethical issues. For instance, to continuously collect data from the user, developers of a digital twin for personalized health care services would need to address worries about surveillance and issues related to data accessibility. However, even if the developers properly deal with these issues, ill-designed algorithms might still cause great harm to users of the digital twin for personalized health care services by offering them a distorted picture of a person’s health conditions. In a situation where the digital twin for personalized health care services is free from data-related concerns such as data collection and data analysis, the service could still induce negative influences on its users by taking an overly demanding concept of health as the norm (coercive healthism).

This process-oriented framework shows that although many ethical concerns are interrelated, they might arise independently in different phases. Breaking down a digital twin for personalized health care service into 4 major phases helps developers of this service conduct an ethical assessment during the design stage in a more systematic way.

**Table 2:** A process-oriented ethical map.

Operation process and operational problem		Ethical issues
**Data collection**		
	Hypercollection	Autonomy Informed consent Right to privacy Surveillance health care
	Data quality and unorthodox use	Distortion of the understanding of health
**Data management**		
	Data ownership and data accessibility	Autonomy Health equity
	Data ownership and data brokerage	Autonomy or informed consent Right to privacy Transparency
	Hacking	Right to privacy
**Data analysis**		
	Biased algorithms	Discrimination or injustice Distortion of the understanding of health
	Biased training data set	Discrimination or injustice Distortion of the understanding of health
**Information use**		
	Decontextualization of disease formation	Autonomy Distortion of the understanding of health Victim blaming
	Epistemic injustice	Autonomy Distortion of the understanding of health Damage physician–patient relationship
	Overdiagnosis	Distortion of the understanding of health Right to bodily integrity

#### Data Collection

##### Overview

Data collection is an indispensable phase of any digital twin for personalized health care service. All data analyses and simulations require initial data input. However, the potential for gaining more information about a person also exposes the person to several ethical risks. On the normative side, practices such as hypercollection can severely infringe on the *right to privacy* and *autonomy*. With no clear understanding of the scope of data collection, meaningful *informed consent* is often missing. As a service that aims to provide better personalized health care, digital twins for personalized health care services also face several ethical risks from the epistemic side. The quality of the collected data might not be good enough to achieve the desired goals, such as providing a more comprehensive understanding of a person’s health conditions or making an accurate prediction of a person’s likelihood of developing certain diseases.

##### Hypercollection

To construct proper models for personalized analysis, a digital twin for personalized health care service might need to access various data sets to train and recalibrate the algorithms used for data analysis. From an engineering perspective, health-related data can be defined as any data that can contribute to drawing inferences on a person’s health condition. It might be tempting to incorporate data about one’s social media use, education, occupation, and other sources that are not traditionally viewed as health-related data in a digital twin [19]. However, there is also a growing concern that service providers might secretly exploit the data collection process by collecting as much data as possible, although some of the collected data are not relevant to the service the digital twin for personalized health care service aims to provide [15].

Furthermore, even if a digital twin for personalized health care service only requests data that fall under the traditional understanding of health data (eg, electronic health records and biopsy), the developer still has to justify the necessity of including the requested health data. Health data are widely considered highly sensitive. Physicians and health care organizations as patients’ fiduciaries have special moral duties to promote patients’ well-being and protect patients’ privacy [17]. Legally speaking, health data are also subject to stringent legal protection [20]. To access a particular set of health data, one must provide a strong reason to justify why the set of health data is necessary for the task and proactively request informed consent from the patients.

In addition, requesting extensive data from the users might also put the users under undue risks of inference attack, a data mining technique that uses authorized data to access authorized information via inference and common knowledge [21]. The requested data can be used to reveal information that users do not wish to share with the developers, seriously infringing on the users’ right to privacy. Merely stating that inclusion may enhance the predictive power and accuracy of a digital twin for personalized health care services is not sufficient to outweigh the privacy concerns.

The growing accessibility of wearables and biosensors offers developers of digital twin systems opportunities to build up a system that can update a person’s digital twin in real time. The pharmaceutical company, Otsuka, has developed a new generation of digital pills (Abilify MyCite) that helps patients track medicine intake by sending signals to the patient’s mobile devices and relevant parties [22,23]. These technologies are usually marketed as innovations that can empower patients by helping them better manage their health conditions (eg, improving adherence). However, many bioethicists cast doubt on this rhetoric. Some physicians might cajole or coerce their patients to take the digital pills so that they can *monitor* their patients [24,25]. Despite being informed, patients might not truly consent to be monitored by taking ingestibles. Circumstances such as this could increase patients’ anxiety levels and reduce trust between physicians and their patients [26].

##### Data Quality and Unorthodox Use

Another issue related to data collection is data quality and accuracy. Indeed, wearables now make the collection of a wide range of biosignals possible. However, the accuracy of the devices used for data collection varies. Consider the Apple Watch as an example. Despite the increasing interest in incorporating this device into the digital twin ecosystem, a recent review on the accuracy of the Apple Watch’s performance in measuring heart rate and energy expenditure found that although the device offers clinically reliable measurement of heart rates, it systematically overestimates the expenditure of energy in patients with cardiovascular disease [27]. Marcus [28] also pointed out that the false-positive rates were unacceptably high in an Apple-sponsored research on atrial fibrillation. Only 35% of the research participants who participated in the validation phase (n=450) presented with atrial fibrillation when examined by a traditional electrocardiogram.

These studies show that although non–medical-level wearables offer an affordable way for the general public to trace and manage their lifestyle, the accuracy of the data gathered by these devices does not always meet the clinical standards. Instead of paving the path for a more personalized health care service, attempts to capitalize data collected from commercial-level wearables might risk creating a distorted digital image of people. Developers must carefully consider the level of data accuracy required for the services they are developing.

The reliability of a digital twin is also vulnerable to unorthodox use of the service. A user of the digital twin might not follow the instructions properly and therefore compromise the quality or accuracy of the data collected by the device. Some users might deliberately use the device in an unorthodox way to *trick the system* in certain circumstances. For instance, a digital twin for personalized health care service devised by insurance companies could be compromised as some users might be more interested in getting a lower premium rather than tracking how a newly adopted healthy lifestyle could improve their health with the digital twin [29]. If a compromised digital twin is to be linked to other general medical services, the compromised digital twins might also undermine a clinician’s capability to make sound clinical judgment. Developers must take precautionary steps to minimize such risks.

#### Data Management

##### Overview

Developers may devise very different management strategies to optimize their services. However, the differences in management strategies can also create obstacles to data accessibility, diminishing users’ *autonomy* in terms of seeking the best use of their data as they see fit. Certain providers might also engage with data brokerage and sell the entrusted data for profits. Although data brokerage is not inherently unethical, selling sensitive health-related data without explicit consent fails to show due respect for the *right to privacy*. The complicated ICT ecosystem of digital twins for personalized health care services might also expose users to undue hacking risks.

##### Data Accessibility

Digital obsolescence may affect people’s ability to reuse their data for other health care services should the service provider fail to devise proper management strategies after each system upgrade [30]. In addition, it is foreseeable that some of the developers of digital twins for personalized health care services would face a close-down and cease to offer service maintenance thereafter. The disruption of service might create difficulties for users of the digital twins for personalized health care services to retrieve the health-related data they entrusted to the service providers. The fail-fast culture of technology startups might exacerbate this problem. Given that digitalization of health care is an unstoppable trend, the inability to access one’s data would severely affect the quality of health care a person can receive. It is important to recognize these accessibility issues and devise means that allow the users of digital twins for personalized health care services to access, retrieve, and transfer the data they have entrusted to their service provider.

##### Data Brokerage

Despite a lack of consensus on how to characterize data ownership and whether the right to data ownership exists [31], data brokerage as a business model is prevalent in the mobile health industry [32]. Health-related data such as patient experience, medical history, and symptoms are especially valuable to pharmaceutical companies and marketing organizations as they may improve drug development and marketing strategies [15,33]. Although data brokerage as a business model is not inherently unethical, many service providers fail to obtain explicit informed consent from their users. Huckvale et al [34] recently found that of the 36 top-ranked Android and iOS apps for depression and smoking cessation, 29 apps transmitted the entrusted data to Facebook or Google (sometimes both) for advertising and analytics services. Only 12 apps accurately disclosed this practice. Given the levels of sensitivity of health-related data, selling them without obtaining explicit consent from the users might severely affect the users’ right to privacy. Service providers ought to convey their plans, if any, for the secondary use of the entrusted data to relevant parties transparently and seek explicit informed consent from the users.

##### Hacking

The digitalization of health care has also attracted the attention of malicious hackers [35]. A survey conducted by KPMG [36] also showed that 81% of the 223 surveyed organizations experienced cyberattacks. In another study conducted by the Institute for Critical Infrastructure Technology [37], it was estimated that >110 million patients in the United States had their health data compromised in 2015 alone. Given that the promise of digital twins for personalized health care services is built on extensive health-related data, they might attract even more cyberattacks than other services in the health care sector have ever undergone. Developers of digital twins for personalized health care services must invest in cybersecurity to properly safeguard the data entrusted to them and the operation of the systems.

#### Data Analysis

##### Overview

Data processing is an essential phase for extracting and synthesizing information from otherwise fragmented and uninformative data. Well-designed algorithms can reveal valuable information that can enhance decision-making capacity. However, this power also makes algorithms become a double-edged sword—they can be used to crunch accessible data to reveal unauthorized information, posing a great threat to people’s right to privacy. The human tendency to trust automatic systems may make users of a digital twin susceptible to harm brought about by biased algorithms.

##### Biased Algorithms

Algorithms are the backbone of any data-driven health care service. They execute the instruction designed by human developers, sort and weigh various data, and produce the desired information such as risk assessment and prognosis. Although algorithms are not liable to influences such as emotions and fatigue, they could still yield unanticipated discriminatory results. Obermeyer et al [38] recently discovered that Black patients were systematically discriminated against by a widely adopted health care algorithm for identifying patients who are highly likely to need complex health care. The algorithm unintentionally discriminated against Black patients by assigning them lower risks as it used health care costs as a proxy for prediction. It is generally true that the more complex the health needs, the higher the cost. However, using health care costs as a proxy overlooks the fact that expenditure depends partially on health care access. The lower amount of health expenditure observed in Black patients does not imply that they are less ill than White patients. Instead, it is more likely to result from unequal access to health care. Obermeyer et al [38] also found that once replaced by the inappropriate proxy used by the system, patients with African backgrounds could have received additional support from 17.7% to 46.5%. This study shows that developers of a digital twin for personalized health care services must pay extra attention to calibrating and validating the algorithms used in the system.

##### Biased Training Data Set

A digital twin for personalized health care services might incorporate advanced computing technologies such as deep learning and machine learning in the data analysis phase. The powerful technologies can be used to detect hidden correlations between different variables, assisting the digital twin in predictive analysis for health risk assessment or treatment outcome assessment. However, the reliability of deep learning and machine learning can be severely compromised if the data sets used to train these algorithms do not properly reflect the environment in which these algorithms are to navigate [39]. Liu et al [40] found that IBM’s Watson for Oncology was less effective and reliable when applied to non-Western populations as the imagery data used for training Watson were primarily from the Western population. Recently, it was also found that certain data sets used for training machine learning algorithms are, in fact, unfit for the task. The labeling of the chest X-ray images in the ChestXray14 database were not standardized and sometimes did not match with the image at all [41]. Similar problems were also identified in machine learning research that aimed to capitalize on chest radiographs and computed tomography scans to detect COVID-19 [42]. Developers must ensure that the training data reflects the characteristics of the served population and are correctly and consistently labeled. Otherwise, the predictive analysis offered by the digital twin can be misleading and even discriminatory, bringing more harm than benefit to the users.

#### Information Use

##### Overview

The use of health-related information is not a value-free practice. The decision regarding which information is worth presenting conveys the values upheld by the developers of a digital twin for personalized health care services. In the context of predictive analysis, the risk scores a digital twin gives to an individual reflect the conception of health and disease for the developers of the digital twin. Without careful reflection, the developers of the digital twin risk passing down problematic values such as victim-blaming culture and distrust of personal experiences. The goal of earlier diagnosis and intervention could also lead to overdiagnosis and infringement of people’s bodily integrity.

##### Decontextualization of Disease Formation

A digital twin for personalized health care services might overly individualize health issues and overlook the fact that socioenvironmental determinants, such as air pollution, water pollution, and lack of education, also contribute to health problems [43,44]. Victims of environmental pollution and social injustice might be wrongfully blamed for their poor health. In addition, although a digital twin for personalized health care service may allow people to access health information they otherwise could not access, the epistemic improvement does not warrant empowerment. People with lower socioeconomic backgrounds might not know how to use the provided information or not have the agency to act upon the information because of external constraints [45]. Contrary to the goal of empowerment, the digital twin for personalized health care services might burden patients with a sense of powerlessness, guilt, and anxiety. It is especially so for a digital twin for personalized health care service that aims to introduce early interventions in lifestyle diseases such as diabetes, hypertension, and obesity [46]. Users who fail to take the advised change could be accused of being irresponsible about their health (victim blaming).

##### Epistemic Injustice

The growing reliance on health information produced by digital twins for personalized health care services could also lead to undervaluing patients’ personal views and experiential knowledge. Some might think that health information offered by the digital twin is more reliable than a patient’s personal account as the information results from an *objective fact*. However, this view overlooks the fact that this information was generated from a system developed with a human’s limited understanding of human biology and other relevant fields. The information would be full of human interpretation and subject to various biases as well. Rich et al [47] recently found that the discrepancies between the analysis by fitness apps and users’ subjective feelings support the concern over epistemic injustice. Downplaying the patient’s experiential knowledge simply because this piece of knowledge has subjective elements is deeply problematic. Instead of offering a more holistic understanding of health, the digitalization of health could create a distorted understanding of health [15,48].

##### Overdiagnosis

Another concern related to the definition of health is overdiagnosis. One of the general goals of digital twins for personalized health care services is to provide early warnings to its users and assist in preventive health care. However, in practice, early action sometimes leads to overdiagnosis and overtreatment. This sort of ethical dilemma has been highlighted in the personalized medicine literature on the use of biomarkers [49-51]. For example, many bioethicists and clinicians are concerned that genetic testing that can be used to detect *BRCA1* and *BRCA2* mutations might cause overtreatment [52,53], causing harm to a patient’s bodily integrity.

Furthermore, when gaining access to more information about various health-related parameters, it is important to reflect on the extent to which deviations from *the norm* can be considered diseases. Sexuality is a prominent example of this. Hormone levels can also differ significantly between women with and without pregnancies [54]. The conceptual link to nonbinary concepts such as dysfunction, harm, and risk also suggests that there is no clear line to be drawn between diseased and nondiseased states. Following this observation, Walker and Rogers [49] argue that *overdiagnosed cases* can be understood as *borderline cases* that are neither diseased nor healthy but in between. Treating borderline cases and those that are clearly diseased in the same way is morally problematic. Recent advocacy of renaming low-risk conditions that are unlikely to develop into cancers echoes this concern [55]. There is also a growing number of bioethicists and medical practitioners casting doubt on the utility of detecting borderline cases [50]. Without careful stratification and selection of reference groups, a digital twin for personalized health care services might risk providing wrongful health advice to its user. Therefore, developers of a digital twin for personalized health care services ought to consult clinical practitioners and relevant researchers to fine-tune the system with comprehensive epidemiological knowledge.

## Discussion

### Limitations of the Study

The analysis we performed in this research offers a clear overview of the major operational problems that might damage vital ethical values during each of the data processing and information use stages. However, this ethical analysis of digital twins for personalized health care services has several limitations. First, as digital twins for personalized health care services are still in their infancy, the literature directly addressing digital twins for personalized health care services is scarce. Most of the ethical analyses we conducted here is based on the literature in fields that we considered closely linked to digital twins for personalized health care services. However, mapping the major ethical risks in this way renders the analysis heavily influenced by our prior knowledge, and we might have overlooked certain ethical risks of digital twins for personalized health care services. Second, as the process-oriented framework we proposed in this research aims to provide a conceptual map to help developers proactively examine potential ethical risks that might occur in each of the major data processing phases, the framework would be less effective in facilitating developers to examine ethical risks based on the type of information provided by a digital twin for the health care system. For instance, it is a known ethical risk that genetic information can be used to infer the health conditions of a person’s family members. For people who do not want to know whether they are at risk of certain genetic diseases, their right not to know might be infringed by the family member who decided to use the information [56,57]. However, because of the structure of the framework, there is no room for this important discussion. It is desirable to see further research on health information generated by a digital twin for personalized health care services and the associated ethical risks. Third, there could be novel ethical risks of digital twins for personalized health care services that are distinctively different from the concerns that have been identified in the literature on digital health, personalized medicine, and precision medicine. This paper is by no mean trying to provide a definitive account of the ethicality of digital twins for personalized health care services. Further empirical ethics research on digital twins for personalized health care services is necessary to identify such novel ethical issues. For instance, researchers may consider adopting the embedded ethics approach proposed by McLennan et al [58] to investigate the ethicality of digital twins for personalized health care services with developers and stakeholders. For researchers interested in developing ethical guidance for emerging digital twin applications, the ethics parallel research approach advocated by Jongsma and Bredenoord [59] is also worth adopting.

### Conclusions

The concept of digital twins can be applied to a wide variety of personalized health care services. The diversity of digital twins for personalized health care services not only manifests in the sort of health care services they aim to provide but also in the ethical risks they might face. To capture these nuances, we conducted a process-oriented ethical analysis to examine the ethical risks that could appear during data processing and information use. The 10 operational problems and relevant ethical values have been structured with a clear, logical flow. This process-oriented ethical map allows developers of digital twins for personalized health care services and stakeholders to have a comprehensive overview of major ethical risks when refining the design of the digital twin. The ethical values section on the map also helps developers better understand the values they ought to consider when developing solutions for an operational problem they might encounter.

## Abbreviations

ICT: information and communication technology
MRI: magnetic resonance imaging
